# Real-life Psychological Experiences and Cardiac Autonomic Physiology among Survivors of a Myocardial Infarction in Midlife

**DOI:** 10.21203/rs.3.rs-7418040/v1

**Published:** 2025-08-26

**Authors:** Viola Vaccarino, Hua She, Lisa Elon, Tené T. Lewis, Yi-An Ko, Gari Clifford, Quiao Li, Nancy Murrah, Lucy H. Shallenberger, Tatum Roberts, Lewam Stefanos, Eric Fan-Lou, J. Douglas Bremner, Paolo Raggi, Arshed Quyyumi, Amit J. Shah

**Affiliations:** Emory University; Emory University; Emory University; Emory University; Emory University; Emory University School of Medicine; Emory University School of Medicine; Emory University; Emory University; Emory University; Emory University; Emory University; Emory University School of Medicine; University of Alberta; Emory University School of Medicine; Emory University

## Abstract

Middle-aged women with ischemic heart disease have a higher burden of psychosocial factors compared with male counterparts. Data on daily life stress and autonomic physiology could help guide targeted interventions. We studied 302 individuals ≤ 61 years of age (129 women and 173 men) recently hospitalized for a myocardial infarction (MI). All underwent a 7-day home monitoring of mood and stress using ecological momentary assessments, with concomitant Holter monitoring for autonomic physiology. Deceleration capacity (DC), a prognostic marker of parasympathetic activity, was the primary autonomic outcome. Across the week of monitoring, women reported higher levels and variability of negative mood and stress than men, with no differences in positive mood. Women also exhibited significantly lower parasympathetic activity (the daily averaged of DC) compared with men. Heart rate variability was also lower. Among women, but not men, daily negative mood was inversely associated with lower DC: for each 1-log unit higher daily negative mood score DC declined 4.5% in women, with no change in men (p=0.02 for interaction). Women with early-onset MI have more parasympathetic withdrawal than men in daily life, especially during moments of negative mood. These results underscore the need for greater attention to psychosocial management in post-MI women.

## Introduction

Despite advances in treatment and prevention, ischemic heart disease (IHD) remains the leading cause of death in women.^1,2^ In recent decades, young and midlife women have shown worse trends than other groups for both incidence and case-fatality of IHD.^3–11^ When compared to men, younger women with IHD continue to have worse morbidity, mortality, and quality of life, despite having less atherosclerotic burden.^12–17^ Such differences are not due to known risk factors, comorbidities, or treatment differences, and remain unexplained.^6,18–20^

Psychological stress is a common experience with complex definitions and measurement modalities.^21–23^ Excessive or prolonged stress can have adverse consequences on cardiovascular health throughout the lifespan, but these effects are exacerbated in individuals with pre-existing IHD compared with those who are low risk.^22,24–26^ Women with IHD, especially young/midlife women, have a higher burden of psychosocial factors compared with male counterparts, and are at higher risk for adverse cardiovascular consequences from these factors.^27^ Our work in the lab has shown that women with early-onset IHD are twice as likely to develop myocardial ischemia with mental stress than men of similar age,^28–30^ especially diffuse ischemia, which is indicative of microvascular disease.^31^ We have also shown that women with IHD manifest more pronounced inflammation and microvascular constriction with in-lab mental stress than men, and these responses predict adverse events in women.^32–35^ While these findings suggest a disproportionate impact of stress in women (especially those with early onset IHD), data remain scarce regarding exposures in daily life in people with IHD.

Capturing stress-related experiences and physiology in a natural setting is particularly relevant for women, who carry a greater psychosocial burden in everyday life (especially midlife women) that may also persist for longer durations than men due to factors like rumination, self-silencing and interpersonal stressors. These aspects are difficult to capture in a lab. There is dearth of data on the everyday experiences of women and men with IHD and their impact on physiology and outcomes. This information could allow the development of novel behavioral interventions aimed at specific psychological pathways of risk.

Recent technologies have opened new opportunities, as low-profile wearable devices now allow to monitor people in their natural contexts. Autonomic activation and movement can be monitored with passive sensors in conjunction with real-time self-reported stress and mood with ecological momentary assessments (EMA).^36^ The latter allow to assess type, frequency, and severity of stressful exposures in the natural context and their association with physiology while accounting for sleep and physical activity. This approach is more scalable to clinical care and future interventions because it is low-burden and does not require a mental stress lab and can help the development of personalized approaches in care. Autonomic measures derived from wearable heart monitors may also provide digital biomarkers of abnormal stress physiology that can be tracked throughout the disease treatment process and provide feedback on treatment response.

In the current study we used EMA coupled with wearable sensing devices for concurrent cardiac autonomic function to compare real-life psychological experiences and ambulatory autonomic physiology between women and men with early onset IHD. We also aimed at examining whether negative psychological experiences during daily life are related to ambulatory autonomic physiology, and whether there are sex differences in these relationships.

## Methods

### Study sample.

Participants were recruited from Emory University-affiliated hospitals throughout metropolitan Atlanta from February 2020 to December 2023. Eligibility criteria included documented hospitalization for type 1 MI within the previous 8 months and age between 18–60 years at the time of MI occurrence. Type 1 MI diagnosis was confirmed through medical record review using standard criteria of elevated troponin levels accompanied by ischemic symptoms and/or electrocardiographic changes or other evidence of myocardial necrosis; the presence of obstructive coronary artery disease (CAD) was not required for inclusion.^37^ Exclusion criteria included severe comorbid medical or psychiatric disorder that could interfere with study results, such as cancer, renal failure, severe uncontrolled hypertension, current alcohol or substance abuse, or schizophrenia; pregnancy or breastfeeding; and current use of immunosuppressant or psychotropic medications other than antidepressants. Potential participants were also excluded if they had unstable angina, acute MI or decompensated heart failure within the week prior to study participation or if they weighed over 450 pounds. All study procedures were performed in accordance with relevant guidelines and regulations. The study received approval from the Emory University Institutional Review Board, and written informed consent was obtained from each participant.

At an initial clinic visit, trained research personnel obtained detailed sociodemographic, medical history, and medication information, and measured weight and height to calculate the BMI. Race was self-reported and categorized into Black or African American vs. all others for analysis. Validated instruments were employed to assess behavioral, social, and health status information. Psychiatric diagnoses were obtained with the Structured Clinical Interview for DSM-IV (SCID),^38^ which provided lifetime diagnostic information for major depressive disorder and posttraumatic stress disorder (PTSD) and was administered by research personnel with specialized training. Depressive symptomatology (past 2 weeks) was evaluated using the Beck Depression Inventory-II (BDI-II),^39^ and current PTSD symptoms (past month) with the PTSD Checklist for DSM-IV.^40^ We also administered the Spielberger’s Anxiety Inventory (20-item state anxiety module),^41^ the Spielberger’s Anger Inventory (15-item state anger module),^42^ the Everyday Discrimination Scale (10-item version) to assess exposure to discrimination or unfair treatment in everyday life,^43^ and the Perceived stress Scale, 4-item version.^44^

Trained personnel abstracted medical records for the index MI including clinical and angiographic data. Obstructive CAD was defined as ≥ 70% lumen stenosis. Severity of CAD was quantified with the Gensini scoring method, which uses a nonlinear point system for degree of luminal stenosis and takes into account prognostic significance by using weighs according to coronary tree location.^45^

### Monitoring of daily stress and mood.

We loaned each participant a smartphone to use unless they wanted to use their own. We collected EMA of everyday stressors and mood states using similar methods described in the National Study of Daily Experiences (NDSE) from the Midlife in the United States Study (MIDUS).^46,47^ The survey was programmed into a smartphone app called RealLife Exp (LifeData LLC, Marion, IN). The multiple-choice survey popped up on the phone 3 times during the day for 7 days at random times, once between 10 AM and 1 PM, and 2 times between 2 PM and 10 PM, with a minimum of 180 minutes break between survey pop-ups. Participants were asked about stressful events that may have occurred in the hour before and about their mood (happiness, sadness, anger, anxiety, loneliness, distress, and calmness) on a scale between 0 (none of the time) and 100 (all of the time). If a stressful moment was reported, participants were asked to describe the nature of the stressor, and the impact of that event with a series of questions querying about negative effects on daily life (how much the event affected daily routine, financial situation, feelings about self, feelings of others about self, physical health, and safety), rumination (how much they thought about the event since it occurred), changes in subjective stress levels after the event, and changes in fatigue level after the event. They were also asked about emotions after the event, including sadness, anger, anxiety, irritation, confusion, and feeling overwhelmed.

Our primary exposure was the daily mean of momentary ratings of negative mood, including ratings of sadness, anger, anxiety, loneliness, and distress, which were collected 3 times per day on a scale from 0 (none of the time) to 100 (all of the time). We calculated an average negative mood score (range, 0 to 100) as the daily mean of negative mood ratings averaged across the 7-day monitoring period; this method allowed us to include participants who did not complete all 7 days of monitoring. We examined positive mood in a similar manner; a positive mood score was computed as the weekly average of the daily mean of positive mood ratings (happiness and calmness) which were collected in a similar fashion. As measures of variability, we used the standard deviation of negative and positive mood scores calculated across the week.

We also evaluated stressor frequency as the daily percent of pop-up surveys with at least one reported stressor)^48^ which was then averaged across the week, as well as the percent of days with at least one stressor and the percent of at least one moment with reported stress during the monitoring week. As a measure of affective reactivity, we compared negative mood ratings between moments with stress and moments without stress within person.^48–50^

### Monitoring of cardiac autonomic physiology.

We employed an ECG monitor to measure daily autonomic balance and changes in autonomic function with stress in daily life. We used the Bittium Faros^™^ 180L (Bittium Inc., Oulu, Finland), an FDA-cleared, noninvasive, lightweight, water-resistant, ambulatory ECG monitoring patch. This device has been extensively used in research studies and clinically to monitor ECG, heart rate, and heart rate variability (HRV), and is the gold standard for investigating accuracy of other devices.^51–54^ Study staff applied the device on the chest with two electrode patches at the end of the clinic visit and instructed participants to wear it continuously for 7 consecutive days. At the end of this time, participants were instructed to return it using a pre-paid package. ECG data were collected at 250 Hz sampling frequency and 10-bit resolution.^55^ Accelerometer data were collected at 25 Hz. Raw ECG data were analyzed using custom-built validated software,^56,57^ which provided signal quality indices, abnormal rhythm detection, time and frequency domain HRV indices, and DC. Data were segmented in 5-minute rolling windows every 30 seconds. The 5-minute segments were analyzed overall and during stationary periods (to avoid the influence of movement and posture) defined as activity counts below 20 based on the Actiwatch. Data collected during sleep were excluded. Sleep periods were defined automatically by the Actiwatch algorithm based on analysis of the minute-by-minute activity patterns. The quality of the ECG signals was evaluated using our previously validated signal quality index.^58^ Segments with evidence of non-sinus rhythm or poor data were excluded (> 50% data in a 5-minute window below a pre-defined cut-off). This method allowed us to include days where only partial data were available.

Our primary measure of autonomic regulation was daily mean deceleration capacity (DC), a marker of parasympathetic activity and cardiovascular risk,^59^ which can be measured reliably regardless of respiration and activity. Secondary measures were daily mean duration of low DC, defined as ≤ 2.5 ms,^59^ a measure of chronicity of autonomic dysregulation, as well as HRV metrics, including frequency domains [high-frequency (HF) and low-frequency (LF) HRV], and the time domain SDNN (standard deviation of NN intervals), a measure of overall autonomic integration and regulation.^60^ All these measures have been associated with higher total and cardiovascular mortality in the general population and cardiac samples.^59,61^ We examined physiological reactivity as the within person relationship of momentary mood and stress with autonomic function metrics averaged in the hour prior to the EMA prompt.

### Statistical analysis.

Autonomic metrics, average mood scores and the daily percentage of moments of stress were log-transformed. Autonomic metrics were examined overall (all wake moments) and restricting the data to stationary moments only. Because the variable distributions and study results were almost identical, we used the overall data in the analysis. We described demographic, behavioral and clinical characteristics between women and men; no hypothesis testing was done in this descriptive analysis.^62^ Subsequently, we compared psychological experiences using EMA between women and men, using t tests for continuous variables and chi-squared tests for categorical variables. Next, we compared autonomic metrics by sex, before and after adjusting for demographic factors (age and race), clinical risk factors (ever smoking, BMI, history of hypertension, history of diabetes mellitus, left ventricular ejection fraction, and Gensini CAD severity score), medications (beta blockers, ACE inhibitors, ARB medications, statins and antidepressants) and psychiatric history and psychological factors measured at the clinic visit (lifetime history of major depression and of PTSD, Beck Depression Inventory score, and PTSD Symptom Checklist score), using linear regression models.

To examine the relationship between real-time psychological experiences through EMA and ambulatory autonomic physiology we used linear models. Our main predictor was the daily mean of negative mood, averaged across the week. Our main outcome was the daily mean of DC averaged over the week. Using nested models, we sequentially adjusted for baseline variables including sociodemographic factors, cardiovascular risk factors, psychosocial factors, CAD severity indicators and cardiovascular medications. In all models we tested the interaction by sex. The main results were expressed as a percentage difference and 95% confidence interval (CI) in daily means of deceleration capacity per 1-log-unit increase in daily mean negative mood score. To facilitate interpretation, we also presented results based on original units of the negative mood score.

Next, we performed a within-person moment-to-moment analysis using multilevel regression models for repeated measures. The models included a first level for participants, a second level for day, and a third level for EMA prompt, with all the relative interactions, which were dropped if not significant, except for the interaction with sex. In these models, the main independent variable was the negative mood score in that specific moment, and the main dependent variable was DC averaged in the hour prior to the corresponding EMA prompt. A similar analysis was done comparing moments when participants reported stress to moments when no stress was reported. Covariate adjustment in these models was the same as above.

Missing covariate data were minimal (< 4%). Therefore, we did not perform multiple imputations. All analyses were conducted using SAS statistical software version 9.4 (SAS Inc., Cary, NC).

### Data availability.

The datasets generated by this research and analyzed in the current study are not publicly available due to the sensitive and confidential nature of the data, but are available from the corresponding author on reasonable request.

## Results

### Study sample.

Of the 306 participants enrolled in the MIMS3 study, 302 (including 129 women) had either EMA data or ambulatory ECG data and were included in the present analysis. Of these, 15 (5%) had EMA data completely missing, 7 (2%) had ambulatory ECG data completely missing, and only 4 (1%) had both sets of data completely missing; participants with partial EMA and ECG monitoring data were retained for analysis. Total or partial missingness for both EMA and ECG data was similar by sex. The weekly average percentage of EMA prompts that were filled over the maximum expected was 74.2% in women (SD, 24.9%) and 75.8% in men (SD, 26.3%). The mean wear time of the Bittium patch was 6.8 days in women (SD, 0.7) and 6.5 days in men (SD, 1.0), and the duration of analyzable ECG data after quality control was 5.2 days in women (SD, 1.9), and 5.0 days in men (SD, 1.9). There was a weak negative correlation between prompt-answering rate and daily mean negative mood (spearman r = −0.22) and no correlation between prompt-answering rate and daily mean stressful moments (spearman r = 0.04). The median time from the date of the index MI to the study visit was 3 months in both groups.

### Baseline Characteristics.

All participants were younger than 61 years, with a mean age of 51 for both women and men. Women were more likely to be Black and less likely to be currently married compared with men (Table 1). Overall, women had a more adverse psychosocial profile than men. Despite having a higher level of education (71% with more than 12 years of education, vs. 66% of men) women had a lower income, with 31% reporting a yearly income < $25,000 compared with 18% of men. The lifetime prevalences of major depression and PTSD were higher in women than in men, but psychosocial scale scores for current symptoms of depression and PTSD were only mildly elevated among women compared with men, as were scores of state anxiety, state anger, everyday discrimination and perceived stress. Women tended to have higher levels of metabolic risk factors, including a higher BMI and a history of hypertension and diabetes mellitus, but the rate of smoking was similar. Medication use was also similar, except for statins that were more often used by men, and antidepressants that were more often used by women.

Women, when compared to men, had evidence for a less severe index MI, including a lower peak troponin level, a higher left ventricular ejection fraction and a lower occurrence of ST-segment elevation MI (Table 1). The severity of CAD prior to any revascularization was also less in women compared to men, as evidenced by a lower Gensini score and a lower prevalence of obstructive CAD and three-vessel disease.

### Sex differences in daily psychological experiences through ecological momentary assessments.

Compared with men, across the week of monitoring women reported higher levels and higher variability of negative mood (the daily mean EMA ratings of sadness, anger, anxiety, loneliness, and distress), but there were no differences in positive mood (the daily mean EMA ratings of happiness and calmness) (Table 2). Women also reported stressful moments more often than men. However, even though 77% of women and 63% of men had at least one moment perceived as stressful during the week, these moments tended to be infrequent in both groups, with a daily percentage of 8.6 stressful moments in women and 5.9 in men (Table 2). Women perceived such moments as having a higher impact on daily routine and fatigue levels compared with men, although the impact of stressful moments on negative emotions was similar to men. Moments perceived as stressful were related to increased negative mood in that same moment both in women and in men (**Supplementary Fig. 1**).

### Sex differences in ambulatory autonomic function.

Across the week of monitoring, the daily average of log-transformed DC was significantly lower in women compared with men, as were HRV frequency domains (HF-HRV and LF-HRV) and the time domain SDNN (Table 3). The daily mean duration of low DC, defined as ≤ 2.5 ms, was also significantly higher in women than in men: 168 minutes (95% CI, 125 to 210) and 100 (95% CI, 63 to 137) minutes, respectively (p = 0.02). Adjustment for sociodemographic factors, clinical characteristics, and medications, as well as psychiatric history and symptom scores for depression and PTSD at the clinic visit did not substantially alter the study estimates although sex differences in DC became not statistically significant (Table 3). Psychiatric history and psychological symptom scores assessed at the clinic visit were not related to DC across the week of monitoring.

### Associations of daily psychological experiences with ambulatory autonomic function.

**Figure 1:**
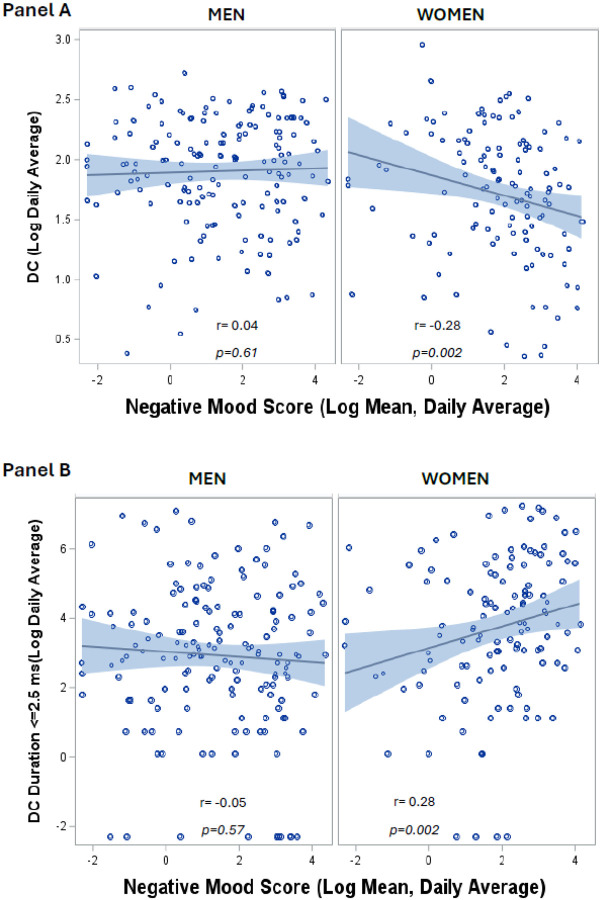
Associations of daily negative mood with ambulatory parasympathetic activity as indexed by deceleration capacity of heart rate (DC). Among women, but not among men, daily life negative mood was negatively associated with daily average level of DC (Panel A) and positively associated with daily average duration of low DC (Panel B).

**Figure 2:**
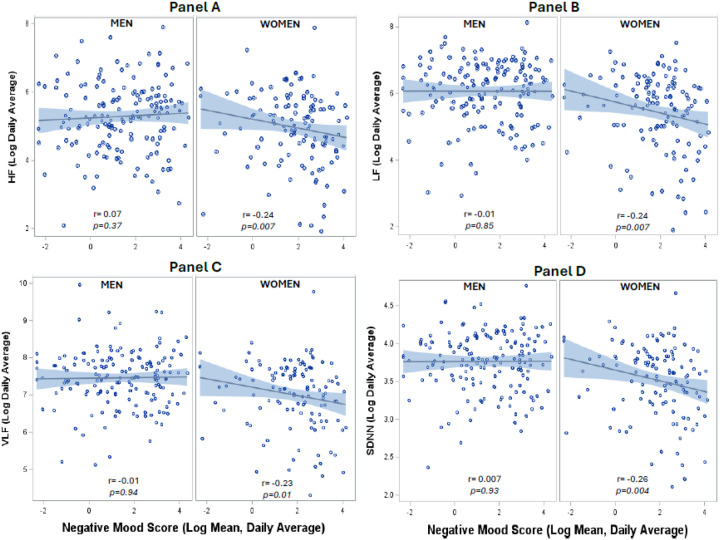
Associations of daily negative mood with ambulatory heart rate variability (HRV). Among women, but not among men, daily life negative mood was negatively associated with the daily average level of HRV metrics, including high-frequency (HF) HRV (Panel A), low-frequency (LF) HRV (Panel B), very-low frequency (VLF) HRV (Panel C) and the standard deviation of NN intervals (SDNN, Panel D).

Among women, but not among men, daily life negative mood was negatively associated with daily average level of DC and positively associated with daily average duration of low DC (Fig. 1). For each 1-log unit higher daily mean negative mood score DC declined 4.5% in women (Table 4) but there was no association among men (p = 0.02 for the interaction with sex). Adjustment for sociodemographic and CAD risk factors, psychosocial factors, and CAD severity in sequential models did not materially affect the associations (Table 4). A similar pattern of results was observed for HRV metrics, which were inversely associated with daily life negative mood in women but not in men (**Supplementary Table 1**, Fig. 2). Expressed with original units, a 20-unit higher daily mean negative mood score was associated with a 12% decline in DC among women, and essentially no change (0.9%) among men (**Supplementary Table 2**). In fully adjusted models results remained similar (**Supplementary Table 3**). Interaction terms for sex were significant in all models.

A within-person moment-to-moment analysis provided consistent results. Moments of negative mood were significantly associated with lower DC averaged in the hour prior the corresponding EMA prompt in the overall population and among women, but not among men, although the interaction with sex was not significant (Table 5). Again, adjustment for the same variables as above in sequential models did not materially affect the associations. In fully adjusted models, among women a 20-unit increase in momentary negative mood score was associated with a statistically significant 2.2% decline in DC in the hour prior to the EMA prompt, while in men the decline was less than 1% and not statistically significant.

In a similar within-person, moment-to-moment analysis, moments with reported stress, compared with moments when no stress was reported, exhibited lower DC averaged in the hour prior to the corresponding EMA prompt (Table 5). The association was similar in women and men. Overall, in a fully adjusted model, there was a 2.3% lower DC (95% CI, −4.1 to −0.6) in the hour prior the corresponding EMA prompt comparing moments with stress to moments with no stress.

## Discussion

We demonstrate important differences in real-life psychological experiences and ambulatory autonomic physiology between women and men with early onset IHD. Using EMA of prospectively sampled experiences in regular life for one week, coupled with wearable sensing devices for cardiac autonomic physiology, women exhibited higher levels and greater variability of negative mood, as well as more moments of perceived stress compared with their male counterparts. Furthermore, women showed on average more autonomic dysregulation than men, as the daily averages of DC and traditional HRV metrics were all lower in women.

Notably, among women, but not among men, daily life negative mood was associated with worse daily autonomic function. Even on a moment-by-moment basis, DC (and other autonomic metrics) was lower in conjunction with moments of more negative mood among women. DC was also lower in conjunction with moments of reported stress, but this association was similar in women and men. These results suggest a unique vulnerability of women with early-onset MI to the influence of negative mood states on autonomic function in daily life, in contrast to the impact of stressful events which is similar by sex. Our results agree with a known excess cardiometabolic risk associated with depression among women.^63,64^ Indeed, depression is a more powerful predictor of major adverse cardiovascular events in women than men, especially at a younger age.^65^

Our results are potentially important to understand the worse morbidity and mortality of younger women with MI compared with men, which have been consistently described despite women having less atherosclerotic vascular disease.^6,18–20^ The cardiac autonomic measures we studied are strong predictors of total and cardiovascular mortality in the general population and cardiac samples.^59,61^ The burden of psychosocial stressors in women is well established,^27^ and recent data have demonstrated women’s susceptibility to adverse cardiovascular responses to laboratory mental stress, including myocardial ischemia,^28–31^ inflammation and microvascular constriction.^32–35^ While these findings suggest a disproportionate impact of stress in women (especially those with early onset IHD), data on psychological exposures in daily life have remained limited. Thus, this study fills a significant gap that may impact current treatment guidelines, as secondary prevention strategies generally focus on management of traditional risk factors and antiplatelet therapies, while little attention is placed on psychosocial or behavioral interventions.^66^

A lab-based mental stress protocol is a rigorous method to study acute stress responses but lacks ecological validity because it does not reflect experiences in everyday life.^67^ Lab-based models rely on the assumption that, even though transient, laboratory responses reflect individuals’ typical stress responses in regular life. However, stress in real life varies in typology, social context, frequency and intensity, all aspects that can differ between women and men.^21,22^ Furthermore, lab-based protocols are resource-intensive and not scalable to real-world clinical settings. The lack of data on everyday experiences in people with IHD has hindered progress toward implementing sex-specific screening modalities and interventions for the prevention and management of IHD.

The reasons for the worse daily life autonomic function in women compared with men require further study. Despite a thorough set of measurements spanning clinical and psychosocial variables obtained at the clinic visit and through medical record review, we were unable to explain the lower autonomic function in daily life among women compared with men. Clinical and psychosocial factors measured at the clinic visit did not contribute to the differences. Women, more than men, met criteria for major depression and PTSD, but these conditions, as also psychosocial scale scores measured at the clinic visit, were not even related to autonomic function during the monitoring week. These results suggest that daily experiences measured prospectively as they occur, and concurrently to autonomic function, are more relevant to uncover risk for women than overall psychosocial burden assessed retrospectively at one point in time.

This study represents the first investigation of sex differences in everyday psychological experiences and their impact on autonomic physiology among individuals with early onset IHD. In addition to evaluating the relationship of psychological experiences and autonomic function prospectively and concurrently, our EMA approach allowed us to examine dimensions of stress and behaviors that are relevant for women (e.g., mood, interpersonal stressors, rumination, impact on daily routine) and that are not easily captured in a laboratory setting. Our study, however, has limitations. We lacked data on subsequent clinical events, and therefore the clinical significance of our findings needs further study. Nonetheless, DC is a recognized intermediate outcome that has been found to predict mortality better than left ventricular ejection fraction.^59^ We cannot exclude selection bias due to the potential exclusion of those who were too ill or otherwise unable to participate; this, however, may have led to underestimation of the true effect size. There could also be selection bias due to missing responses to EMA prompts; however, only weak correlations were found between EMA missingness and daily mood. As our study was conducted at a single institution, the generalizability of our findings to other populations and settings requires further exploration. Furthermore, future studies should examine whether these results apply to all women or are specific to those with early-onset MI. Despite these limitations, this represents the only study to date of daily experiences and their impact on autonomic physiology in middle-aged women and men survivors of MI.

In conclusion, among midlife survivors of a MI, women have a larger burden of negative experiences and more autonomic dysregulation in everyday life than men. Furthermore, daily life negative mood has a larger impact on autonomic physiology in women than in men. These data suggest a unique vulnerability of women with early-onset MI towards the impact of negative mood states on autonomic physiology in daily life. Prospective evaluation of negative experiences as they occur in everyday living could be important to uncover cardiovascular risk among women and could help design sex-specific secondary prevention strategies.

## Supplementary Material

Supplementary Files

This is a list of supplementary files associated with this preprint. Click to download.

- SupplementaryMaterial.final.docx

## Figures and Tables

**Table 1.** Characteristics of the study population by sex.

	Women N=129	Men N=173
**Demographic Factors**		
Age, years, mean (SD)	51 (7)	51 (7)
Black or African American	69	42
Married or living with partner, %	41	58
Education >12 years	71	66
Income < $25,000, %	31	18
Premenopausal, %	38	--
**Psychosocial Risk Factors**		
Lifetime history of major depression, %	42	26
Beck Depression Inventory, mean (SD)	11 (9)	10 (9)
Lifetime history of PTSD, %	24	15
PTSD Symptom Checklist, mean (SD)	32 (13)	28 (12)
State Anxiety, mean (SD)	36 (12)	33 (12)
State Anger, mean (SD)	19 (9)	18 (6)
Everyday Discrimination Scale, mean (SD)	18 (6)	17 (7)
Perceived Stress Scale	6 (4)	5 (4)
**Cardiovascular Risk Factors and Health Behaviors**		
BMI, kg/m^2^, mean (SD)	33 (6)	30 (5)
Systolic blood pressure, mmHg, mean (SD)	136 (20)	130 (19)
Diastolic blood pressure, mmHg, mean (SD)	84 (12)	81 (12)
Heart rate, beat/min, mean (SD)	67 (11)	62 (12)
History of hypertension, %	81	75
History of dyslipidemia, %	69	78
History of diabetes, %	40	32
Ever smoker, %	40	41
Habitual physical activity (Baecke score)	7 (1)	7 (1)
**Medications**		
Beta Blocker, %	85	87
Statins, %	83	92
Aspirin, %	87	88
ACE Inhibitors, %	20	34
ARB medications, %	36	23
Antidepressants, %	22	9
**Clinical Characteristics**		
Maximum troponin, ng/mL, mean (SD)	6 (11)	13 (20)
LV ejection fraction (%), mean (SD)	52 (12)	48 (12)
ST-segment elevation MI, %	18	38
Gensini CAD severity score, mean (SD)*	20 (5)	40 (3)
Obstructive CAD (stenosis ≥70%), %	73	92
Three-vessel disease (at ≥70%), %	5	12
History of congestive heart failure, %	9	5
History of MI prior to index MI, %	20	19
History of revascularization, %	84	95

SD: standard deviation; BMI: Body mass index; ACE: angiotensin-converting enzyme; ARB: angiotensin receptor blocker; MI: myocardial infarction; LV: left ventricle; CAD: coronary artery disease.

* Geometric means.

**Table 2:** Sex differences in mood states and stress during ecological momentary assessments across the week of monitoring.

	Women N = 123	Men N = 164	P
**Negative mood**, geometric mean (SD)			
Negative mood score^a^	6.2 (4.2)	4.1 (5.5)	0.02
Negative mood variability^b^	3.9 (3.1)	2.5 (3.9)	0.003
**Positive mood**, geometric mean (SD)			
Positive mood score^a^	69.1 (1.5)	67.0 (1.7)	0.95
Positive mood variability^b^	7.2 (2.8)	6.3 (2.9)	0.31
**Stress**			
Daily percent of moments with stress, geometric mean (SD)	8.6 (3.8)	5.9 (4.3)	0.02
Percent of days with at least one stressor, geometric mean (SD)	8.2 (11.7)	3.9 (17.4)	0.02
At least one moment with stress during the week, n (%)	95 (77.2)	103 (62.8)	0.009
**Impact of moments with stress on life and emotions** ^c^			
Negative effects on daily life, mean (SD)^d^	32.4 (23.3)	25.6 (18.9)	0.03
Rumination, mean (SD)	52.2 (30.6)	44.2 (31.4)	0.07
Change in subjective stress level, mean (SD)	60.7 (26.3)	45.7 (26.3)	< 0.001
Change in fatigue level, mean (SD)	32.6 (26.0)	24.5 (24.4)	0.05
Within-person difference in negative mood score between moments with perceived stress and moments with no perceived stress (95% CI)^e^	14.9 (13.4, 16.4)	10.3 (8.9, 11.7)	< 0.001
Within-person difference in positive mood score between moments with perceived stress and moments with no perceived stress (95% CI)^e^	−14.8 (−17.2, −12.4)	−15.4 (−17.7, −13.1)	0.74

SD: standard deviation; CI: confidence interval.

a Daily mean of mood ratings on a scale from 0 (none of the time) to 100 (all of the time), averaged across moments, and then averaged across the week of monitoring.

b Daily mean of standard deviation of mood ratings, averaged across moments, and then averaged across the week of monitoring.

c Among those who reported at least one moment with stress (95 women and 103 men).

d Weekly mean scores summing separate questions (asked after a stressful moment), each on a scale from 0 to 100: whether the event affected daily routine, financial situation, feeling of self, feeling of other people about self, and physical health or safety.

e Estimates from a repeated measures model. The mood scores are on a scale from 0 to 100.

**Table 3:** Sex differences in autonomic function across the week of monitoring.

Autonomic function metric^a^	Women N = 127	Men N = 168	P
	**Unadjusted Means (95% CI)**		
Ln DC	1.7 (1.6, 1.8)	1.9 (1.8, 2.0)	0.01
Ln HF	5.0 (4.8, 5.2)	5.3 (5.1, 5.4)	0.03
Ln LF	5.5 (5.3, 5.7)	6.0 (5.9, 6.2)	< 0.001
Ln VLF	7.0 (6.9, 7.2)	7.4 (7.3, 7.6)	< 0.001
Ln SDNN	3.5 (3.5, 3.6)	3.8 (3.7, 3,8)	< 0.001
	**Means Adjusted for Sociodemographic and Clinical Factors (95% CI)** ^b,c^		
Ln DC	1.8 (1.7, 1.8)	1.9 (1.8, 1.9)	0.10
Ln HF	4.9 (4.8, 5.1)	5.3 (5.1, 5.5)	0.01
Ln LF	5.5 (5.3, 5.7)	6.0 (5.9, 6.2)	< 0.001
Ln VLF	7.1 (6.9, 7.2)	7.4 (7.3, 7.5)	< 0.001
Ln SDNN	3.5 (3.5, 3.6)	3.7 (3.7, 3.8)	< 0.001
	**Means Further Adjusted for Psychological Factors (95% CI)** ^d,e^		
Ln DC	1.8 (1.7, 1.9)	1.9 (1.8, 1.9)	0.13
Ln HF	5.0 (4.8, 5.1)	5.3 (5.1, 5.5)	0.02
Ln LF	5.5 (5.3, 5.7)	6.0 (5.9, 6.2)	< 0.001
Ln VLF	7.1 (6.9, 7.2)	7.4 (7.3, 7.5)	0.002
Ln SDNN	3.5 (3.5, 3.6)	3.7 (3.7, 3.8)	< 0.001
	**Unadjusted Means (95% CI)**		

CI: confidence interval; DC: deceleration capacity; HF: high-frequency heart rate variability; LF: low-frequency heart rate variability; VLF: very-low frequency heart rate variability; SDNN: standard deviation of NN intervals.

a Daily means averaged across the week.

b Adjusted for sociodemographic factors (age, race (Black versus non-Black), married, education > 12 years), clinical risk factors (ever smoking, BMI, history of hypertension, history of diabetes mellitus, left ventricular ejection fraction, Gensini CAD severity score) and medications (beta blockers, ACE inhibitors, ARB medications, statins and antidepressants).

c 8 participants not included due to missing values.

d Further adjusted for psychiatric history (lifetime history of major depression and of PTSD), and for Beck Depression Inventory score, and PTSD Symptom Checklist score at the clinic visit.

e 14 participants not included due to missing values.

**Table 4:** Sex differences in associations of daily average negative mood and daily average ambulatory autonomic function as indexed by deceleration capacity of heart rate.

	Women N = 121	Men N = 159	
	**Percent difference (95% CI) in daily means of ln DC per 1-log-unit increase in daily mean negative mood score**		**P for sex interaction** *
Unadjusted	−4.5 (−7.8, −1.2)	0.5 (−2.0, 2.9)	0.02
Adjusted for sociodemographic factors*	−3.8 (−6.8, −0.9)	0.3 (−2.0, 2.5)	0.03
Adjusted for the above plus CAD risk factors†	−3.6 (−6.2, −1.1)	0.7 (−1.3, 2.6)	0.009
Adjusted for the above plus psychosocial scales‡ and antidepressant medication use	−3.5 (−6.2, −0.8)	0.9 (−1.2, 3.0)	0.007
Adjusted for the above plus CAD severity¶	−4.3 (−7.3, −1.3)	0.8 (−1.5, 3.1)	0.005
Adjusted for the above plus cardiovascular medications ⊠	−4.2 (−7.1, −1.2)	0.6 (−1.7, 3.0)	0.008

CI: confidence interval; DC: deceleration capacity; CAD: coronary artery disease.

* Age, sex (in overall models), race (black versus non-black), married, education > 12 years.

† Ever smoking, BMI, history of hypertension and diabetes mellitus.

‡ Beck Depression Inventory score, and PTSD Symptom Checklist score at the clinic visit.

¶ Left ventricular ejection fraction, Gensini CAD severity score, ST-segment elevation MI.

⊠ Beta-blockers, angiotensin-converting enzyme inhibitors, angiotensin receptor blockers, and statins.

**Table 5:** Within-person association between moments of negative mood or perceived stress and deceleration capacity of heart rate averaged in the hour prior the corresponding EMA prompt.

	Overall	Women	Men
	**Percent difference (95% CI) in ln DC per 20-unit increase in momentary negative mood score**		
Unadjusted	−1.8 (−3.0, −0.5)	−2.2 (−3.9, −0.4)	−1.3 (−3.1, 0.5)
Adjusted for sociodemographic factors*	−1.4 (−2.5, −0.4)	−1.8 (−3.3, −0.3)	−1.2 (−2.7, 0.4)
Adjusted for the above plus CAD risk factors†	−1.3 (−2.2, −0.3)	−1.6 (−2.9, −0.3)	−1.0 (−2.4, 0.5)
Adjusted for the above plus psychosocial scales‡ and antidepressant medication use	−1.2 (−2.1, −0.2)	−1.6 (−2.9, −0.3)	−0.7 (−2.1, 0.7)
Adjusted for the above plus CAD severity¶	−1.4 (−2.5, −0.3)	−1.9 (−3.4, −0.3)	−0.9 (−2.6, 0.7)
Adjusted for the above plus cardiovascular medications ⊠	−1.5 (−2.7, −0.4)	−2.2 (−3.8, −0.6)	−0.9 (−2.3, 0.8)
	**Percent difference (95% CI) in ln DC comparing moments with stress to moments with no stress**		
Unadjusted	−2.5 (−4.5, −0.6)	−2.0 (−5.1, 1.0)	−2.9 (−5.5, −0.3)
Adjusted for sociodemographic factors*	−2.2 (−3.9, −0.5)	−1.8 (−4.4, 0.9)	−2.6 (−5.0, −0.3)
Adjusted for the above plus CAD risk factors†	−2.0 (−3.5, −0.4)	−1.7 (−4.1, 0.7)	−2.4 (−4.5, −0.2)
Adjusted for the above plus psychosocial scales‡ and antidepressant medication use	−1.9 (−3.4, −0.4)	−1.8 (−4.1, 0.5)	−2.1 (−4.1, 0.01)
Adjusted for the above plus CAD severity¶	−2.2 (−4.0, −0.5)	−2.1 (−4.8, 0.5)	−2.5 (−4.9, −0.1)
Adjusted for the above plus cardiovascular medications⊠	−2.3 (−4.1, −0.6)	−2.3 (−4.9, 0.4)	−2.5 (−4.9, −0.1)

CI: confidence interval; DC: deceleration capacity; CAD: coronary artery disease.

* Age, sex (in overall models), race (black versus non-black), married, education > 12 years.

† Ever smoking, BMI, history of hypertension and diabetes mellitus.

‡ Beck Depression Inventory score, and PTSD Symptom Checklist score at the clinic visit.

¶ Left ventricular ejection fraction, Gensini CAD severity score, ST-segment elevation MI.

⊠ Beta-blockers, angiotensin-converting enzyme inhibitors, angiotensin receptor blockers, and statins.

## Data Availability

The datasets generated by this research and analyzed in the current study are not publicly available due to the sensitive and confidential nature of the data, but are available from the corresponding author on reasonable request.
